# 2-[(5-Methyl­thio­phen-2-yl)methyl­idene]malono­nitrile

**DOI:** 10.1107/S1600536813014487

**Published:** 2013-06-08

**Authors:** Xuewei Liu, Zhengbang Chen, Weiwei Cao, Haifeng Gan, Kai Guo

**Affiliations:** a College of Biotechnology and Pharmaceutical Engineering, Nanjing University of Technology, Puzhunan Road No.30 Nanjing, Nanjing 210009, People’s Republic of China

## Abstract

There are two independent molecules in the asymmetric unit of the title compound, C_9_H_6_N_2_S, which is an inter­mediate compound of a cardiovascular drug. The two molecules are nearly planar, displaying dihedral angles of 3.5 (2) and 5.7 (2)° between the thiophene ring and the malononitrile moiety. In the crystal, C—H⋯N inter­actions lead to the formation of a sheet structure that packs in a parallel fashion.

## Related literature

For a related structure, see: Altundas *et al.* (2011 ▶). For bond-length data, see: Allen *et al.* (1987 ▶).
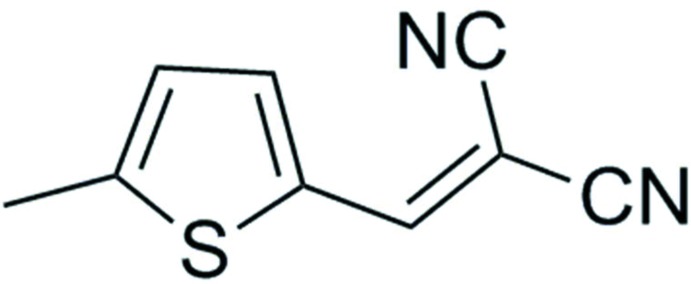


## Experimental

### 

#### Crystal data

C_9_H_6_N_2_S
*M* *_r_* = 174.22
Triclinic, 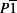

*a* = 9.1120 (18) Å
*b* = 9.9380 (2) Å
*c* = 10.1350 (2) Å
α = 81.10 (3)°
β = 80.71 (3)°
γ = 86.70 (3)°
*V* = 894.3 (3) Å^3^
*Z* = 4
Mo *K*α radiation
μ = 0.30 mm^−1^
*T* = 293 K
0.30 × 0.20 × 0.10 mm

#### Data collection

Enraf–Nonius CAD-4 diffractometer
Absorption correction: ψ scan (North *et al.*, 1968 ▶) *T*_min_ = 0.915, *T*_max_ = 0.970
3465 measured reflections
3247 independent reflections
1399 reflections with *I* > 2σ(*I*)
*R*_int_ = 0.081
3 standard reflections every 200 reflections intensity decay: 1%

#### Refinement

*R*[*F* ^2^ > 2σ(*F* ^2^)] = 0.070
*wR*(*F* ^2^) = 0.102
*S* = 1.00
3247 reflections
217 parameters
H-atom parameters constrained
Δρ_max_ = 0.20 e Å^−3^
Δρ_min_ = −0.28 e Å^−3^

### 

Data collection: *CAD-4 EXPRESS* (Enraf–Nonius, 1989 ▶); cell refinement: *CAD-4 EXPRESS*; data reduction: *XCAD4* (Harms & Wocadlo, 1995 ▶); program(s) used to solve structure: *SHELXS97* (Sheldrick, 2008 ▶); program(s) used to refine structure: *SHELXL97* (Sheldrick, 2008 ▶); molecular graphics: *SHELXTL-Plus* (Sheldrick, 2008 ▶); software used to prepare material for publication: *SHELXL97*.

## Supplementary Material

Crystal structure: contains datablock(s) global, I. DOI: 10.1107/S1600536813014487/ds2229sup1.cif

Structure factors: contains datablock(s) I. DOI: 10.1107/S1600536813014487/ds2229Isup2.hkl

Supplementary material file. DOI: 10.1107/S1600536813014487/ds2229Isup3.cml

Additional supplementary materials:  crystallographic information; 3D view; checkCIF report

## Figures and Tables

**Table 1:** Hydrogen-bond geometry (Å, °)

*D*—H⋯*A*	*D*—H	H⋯*A*	*D*⋯*A*	*D*—H⋯*A*
C3—H3*A*⋯N2^i^	0.93	2.52	3.439 (6)	168
C6—H6*A*⋯N3^ii^	0.93	2.52	3.434 (6)	169
C12—H12*A*⋯N4^iii^	0.93	2.60	3.518 (6)	171
C15—H15*A*⋯N1^i^	0.93	2.51	3.430 (6)	170

Symmetry codes: (i) 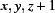; (ii) 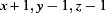; (iii) 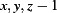.

**Fig. 1.**
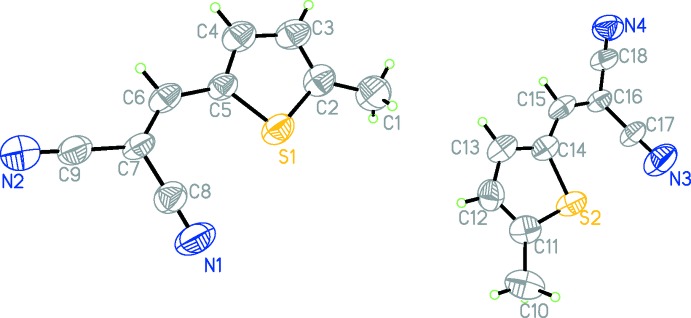
The molecular structure of the title molecule, with the atom-numbering scheme. Fig. 2. A practical packing diagram of the title compound. Hydrogen bonds are shown as dashed lines.

**Fig. 2.**
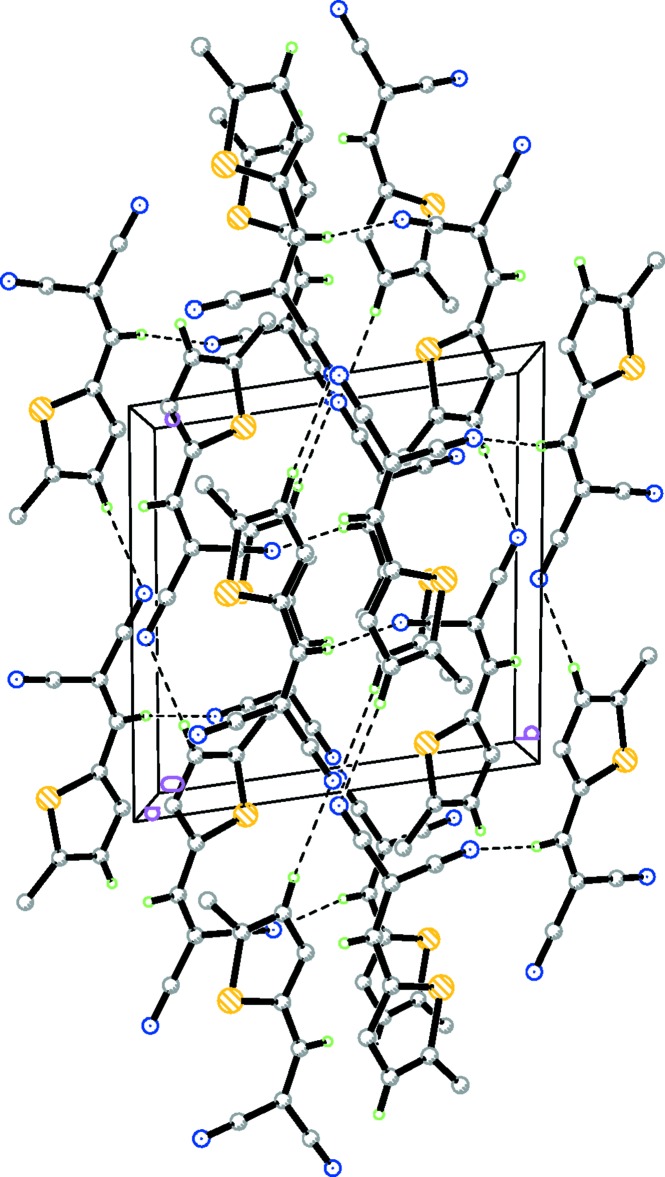
A packing diagram for the title compound.

## supplementary crystallographic information

### Experimental

To a solution of 5-methylthiophene-2-carbaldehyde (10.02 mmol, 1.27 g) and malononitrile (10.13 mmol, 0.67 g) in ethanol (20 ml) was added triethyl (0.31 ml) and the reaction mixture stired at room temperature for 3 h. The reaction solution was filtered to get the title compound (1.44 g) as yellow solid. Crystals of the title compound for X-ray diffraction were obtained by slow evaporation of an acetone solution.

### Refinement

H atoms were positioned geometrically with C—H = 0.93 and 0.96 for aromatic and methyl H atoms, respectively, and constrained to ride on their parent atoms, with *U*_iso_(H) = 1.2 (or 1.5 for methyl groups) times *U*_eq_(C).

### Crystal data

**Table:**

C_9_H_6_N_2_S	*Z* = 4
*M**_r_* = 174.22	*F*(000) = 360
Triclinic, *P*1	*D*_x_ = 1.294 Mg m^−^^3^
Hall symbol: -P 1	Mo *K*α radiation, λ = 0.71073 Å
*a* = 9.1120 (18) Å	Cell parameters from 25 reflections
*b* = 9.9380 (2) Å	θ = 9–13°
*c* = 10.1350 (2) Å	µ = 0.30 mm^−^^1^
α = 81.10 (3)°	*T* = 293 K
β = 80.71 (3)°	Colorless, yellow
γ = 86.70 (3)°	0.30 × 0.20 × 0.10 mm
*V* = 894.3 (3) Å^3^	

### Data collection

**Table:**

Enraf–Nonius CAD-4 diffractometer	1399 reflections with *I* > 2σ(*I*)
Radiation source: fine-focus sealed tube	*R*_int_ = 0.081
Graphite monochromator	θ_max_ = 25.4°, θ_min_ = 2.1°
ω/2θ scans	*h* = 0→10
Absorption correction: ψ scan (North *et al.*, 1968)	*k* = −11→11
*T*_min_ = 0.915, *T*_max_ = 0.970	*l* = −12→12
3465 measured reflections	3 standard reflections every 200 reflections
3247 independent reflections	intensity decay: 1%

### Refinement

**Table:**

Refinement on *F*^2^	Primary atom site location: structure-invariant direct methods
Least-squares matrix: full	Secondary atom site location: difference Fourier map
*R*[*F*^2^ > 2σ(*F*^2^)] = 0.070	Hydrogen site location: inferred from neighbouring sites
*wR*(*F*^2^) = 0.102	H-atom parameters constrained
*S* = 1.00	*w* = 1/[σ^2^(*F*_o_^2^) + (0.017*P*)^2^] where *P* = (*F*_o_^2^ + 2*F*_c_^2^)/3
3247 reflections	(Δ/σ)_max_ < 0.001
217 parameters	Δρ_max_ = 0.20 e Å^−^^3^
0 restraints	Δρ_min_ = −0.28 e Å^−^^3^

### Special details

**Table:**

Geometry. All e.s.d.'s (except the e.s.d. in the dihedral angle between two l.s. planes) are estimated using the full covariance matrix. The cell e.s.d.'s are taken into account individually in the estimation of e.s.d.'s in distances, angles and torsion angles; correlations between e.s.d.'s in cell parameters are only used when they are defined by crystal symmetry. An approximate (isotropic) treatment of cell e.s.d.'s is used for estimating e.s.d.'s involving l.s. planes.
Refinement. Refinement of *F*^2^ against ALL reflections. The weighted *R*-factor *wR* and goodness of fit *S* are based on *F*^2^, conventional *R*-factors *R* are based on *F*, with *F* set to zero for negative *F*^2^. The threshold expression of *F*^2^ > σ(*F*^2^) is used only for calculating *R*-factors(gt) *etc*. and is not relevant to the choice of reflections for refinement. *R*-factors based on *F*^2^ are statistically about twice as large as those based on *F*, and *R*- factors based on ALL data will be even larger.

### Fractional atomic coordinates and isotropic or equivalent isotropic displacement parameters (Å^2^)

**Table:**

	*x*	*y*	*z*	*U*_iso_*/*U*_eq_	
S1	0.49006 (14)	0.26105 (12)	−0.05976 (12)	0.0669 (4)	
N1	0.4196 (5)	0.3320 (4)	−0.3837 (4)	0.0881 (14)	
C1	0.4416 (6)	0.3227 (5)	0.2007 (5)	0.0890 (16)	
H1A	0.4734	0.2980	0.2872	0.133*	
H1B	0.3370	0.3081	0.2092	0.133*	
H1C	0.4603	0.4171	0.1682	0.133*	
C2	0.5250 (5)	0.2376 (5)	0.1038 (4)	0.0659 (14)	
N2	0.7023 (5)	0.0147 (5)	−0.5565 (4)	0.0922 (15)	
C3	0.6315 (5)	0.1367 (5)	0.1227 (5)	0.0712 (15)	
H3A	0.6654	0.1091	0.2047	0.085*	
C4	0.6835 (5)	0.0797 (4)	0.0059 (4)	0.0657 (13)	
H4A	0.7563	0.0102	0.0025	0.079*	
C5	0.6177 (5)	0.1354 (4)	−0.1039 (4)	0.0505 (12)	
C6	0.6545 (5)	0.0882 (4)	−0.2296 (4)	0.0540 (12)	
H6A	0.7237	0.0157	−0.2307	0.065*	
C7	0.6063 (4)	0.1304 (4)	−0.3479 (4)	0.0525 (12)	
C8	0.5028 (5)	0.2431 (5)	−0.3670 (4)	0.0630 (14)	
C9	0.6586 (5)	0.0674 (5)	−0.4633 (5)	0.0644 (14)	
S2	−0.01039 (14)	0.76331 (12)	0.47759 (12)	0.0648 (4)	
N3	−0.0749 (5)	0.8358 (4)	0.8055 (4)	0.1014 (16)	
N4	0.2033 (5)	0.5077 (4)	0.9825 (4)	0.0876 (14)	
C10	−0.0682 (5)	0.8159 (5)	0.2138 (4)	0.0893 (17)	
H10A	−0.0390	0.7866	0.1272	0.134*	
H10B	−0.0479	0.9107	0.2070	0.134*	
H10C	−0.1726	0.8030	0.2428	0.134*	
C11	0.0171 (5)	0.7347 (5)	0.3137 (4)	0.0602 (13)	
C12	0.1235 (6)	0.6340 (5)	0.2928 (4)	0.0652 (14)	
H12A	0.1542	0.6064	0.2088	0.078*	
C13	0.1810 (5)	0.5769 (4)	0.4076 (5)	0.0641 (13)	
H13A	0.2525	0.5063	0.4094	0.077*	
C14	0.1206 (4)	0.6363 (4)	0.5186 (4)	0.0540 (12)	
C15	0.1587 (5)	0.5924 (4)	0.6492 (4)	0.0563 (12)	
H15A	0.2295	0.5211	0.6526	0.068*	
C16	0.1108 (5)	0.6353 (4)	0.7697 (4)	0.0506 (11)	
C17	0.0059 (5)	0.7456 (5)	0.7899 (4)	0.0603 (14)	
C18	0.1617 (5)	0.5647 (5)	0.8883 (5)	0.0640 (14)	

### Atomic displacement parameters (Å^2^)

**Table:**

	*U*^11^	*U*^22^	*U*^33^	*U*^12^	*U*^13^	*U*^23^
S1	0.0696 (9)	0.0566 (8)	0.0684 (9)	0.0139 (7)	−0.0053 (7)	−0.0016 (6)
N1	0.083 (3)	0.074 (3)	0.102 (3)	0.030 (3)	−0.019 (3)	−0.004 (2)
C1	0.095 (4)	0.091 (4)	0.076 (4)	0.006 (3)	−0.007 (3)	−0.006 (3)
C2	0.061 (3)	0.069 (3)	0.061 (3)	0.000 (3)	0.004 (3)	−0.002 (3)
N2	0.088 (4)	0.104 (4)	0.084 (3)	0.024 (3)	−0.023 (3)	−0.013 (3)
C3	0.068 (4)	0.068 (4)	0.070 (3)	0.008 (3)	−0.008 (3)	0.009 (3)
C4	0.071 (4)	0.050 (3)	0.070 (3)	0.006 (3)	−0.010 (3)	0.006 (2)
C5	0.052 (3)	0.045 (3)	0.047 (3)	0.007 (2)	−0.002 (2)	0.007 (2)
C6	0.049 (3)	0.038 (3)	0.066 (3)	0.005 (2)	0.001 (2)	0.008 (2)
C7	0.043 (3)	0.047 (3)	0.061 (3)	0.014 (2)	−0.003 (2)	0.003 (2)
C8	0.059 (3)	0.065 (3)	0.059 (3)	0.008 (3)	0.000 (3)	−0.002 (3)
C9	0.057 (3)	0.066 (3)	0.065 (3)	0.016 (3)	−0.010 (3)	0.000 (3)
S2	0.0640 (9)	0.0575 (8)	0.0679 (9)	0.0153 (7)	−0.0109 (7)	0.0010 (6)
N3	0.118 (4)	0.083 (3)	0.095 (3)	0.038 (3)	−0.005 (3)	−0.014 (3)
N4	0.097 (4)	0.087 (3)	0.079 (3)	0.019 (3)	−0.029 (3)	−0.003 (2)
C10	0.083 (4)	0.110 (4)	0.072 (3)	−0.007 (3)	−0.023 (3)	0.008 (3)
C11	0.058 (3)	0.061 (3)	0.053 (3)	−0.010 (3)	0.002 (2)	0.009 (2)
C12	0.072 (4)	0.065 (3)	0.058 (3)	−0.002 (3)	−0.001 (3)	−0.016 (3)
C13	0.057 (3)	0.054 (3)	0.075 (3)	0.010 (2)	−0.002 (3)	−0.001 (3)
C14	0.048 (3)	0.048 (3)	0.061 (3)	0.003 (2)	−0.001 (2)	−0.001 (2)
C15	0.046 (3)	0.040 (3)	0.077 (3)	0.010 (2)	−0.004 (2)	0.001 (2)
C16	0.054 (3)	0.042 (3)	0.052 (3)	0.010 (2)	−0.007 (2)	0.001 (2)
C17	0.069 (3)	0.046 (3)	0.061 (3)	0.019 (3)	−0.005 (3)	−0.004 (2)
C18	0.054 (3)	0.063 (3)	0.072 (3)	0.018 (3)	−0.013 (3)	−0.006 (3)

### Geometric parameters (Å, º)

**Table:**

S1—C2	1.716 (4)	S2—C11	1.704 (4)
S1—C5	1.717 (4)	S2—C14	1.736 (4)
N1—C8	1.141 (5)	N3—C17	1.141 (5)
C1—C2	1.483 (6)	N4—C18	1.141 (5)
C1—H1A	0.9600	C10—C11	1.486 (5)
C1—H1B	0.9600	C10—H10A	0.9600
C1—H1C	0.9600	C10—H10B	0.9600
C2—C3	1.368 (6)	C10—H10C	0.9600
N2—C9	1.155 (5)	C11—C12	1.369 (6)
C3—C4	1.394 (5)	C12—C13	1.381 (6)
C3—H3A	0.9300	C12—H12A	0.9300
C4—C5	1.375 (5)	C13—C14	1.373 (5)
C4—H4A	0.9300	C13—H13A	0.9300
C5—C6	1.409 (5)	C14—C15	1.420 (5)
C6—C7	1.342 (5)	C15—C16	1.353 (5)
C6—H6A	0.9300	C15—H15A	0.9300
C7—C9	1.414 (6)	C16—C18	1.426 (5)
C7—C8	1.432 (6)	C16—C17	1.429 (5)
			
C2—S1—C5	92.6 (2)	C11—S2—C14	91.3 (2)
C2—C1—H1A	109.5	C11—C10—H10A	109.5
C2—C1—H1B	109.5	C11—C10—H10B	109.5
H1A—C1—H1B	109.5	H10A—C10—H10B	109.5
C2—C1—H1C	109.5	C11—C10—H10C	109.5
H1A—C1—H1C	109.5	H10A—C10—H10C	109.5
H1B—C1—H1C	109.5	H10B—C10—H10C	109.5
C3—C2—C1	129.9 (5)	C12—C11—C10	128.2 (4)
C3—C2—S1	110.8 (4)	C12—C11—S2	111.3 (3)
C1—C2—S1	119.2 (4)	C10—C11—S2	120.5 (4)
C2—C3—C4	112.8 (4)	C11—C12—C13	114.0 (4)
C2—C3—H3A	123.6	C11—C12—H12A	123.0
C4—C3—H3A	123.6	C13—C12—H12A	123.0
C5—C4—C3	114.0 (4)	C14—C13—C12	112.4 (4)
C5—C4—H4A	123.0	C14—C13—H13A	123.8
C3—C4—H4A	123.0	C12—C13—H13A	123.8
C4—C5—C6	121.7 (4)	C13—C14—C15	122.8 (4)
C4—C5—S1	109.8 (3)	C13—C14—S2	111.0 (3)
C6—C5—S1	128.5 (3)	C15—C14—S2	126.1 (3)
C7—C6—C5	130.7 (4)	C16—C15—C14	131.7 (4)
C7—C6—H6A	114.6	C16—C15—H15A	114.1
C5—C6—H6A	114.6	C14—C15—H15A	114.1
C6—C7—C9	121.4 (4)	C15—C16—C18	119.4 (4)
C6—C7—C8	122.8 (4)	C15—C16—C17	124.7 (4)
C9—C7—C8	115.8 (4)	C18—C16—C17	115.8 (4)
N1—C8—C7	178.9 (5)	N3—C17—C16	178.3 (5)
N2—C9—C7	179.2 (5)	N4—C18—C16	179.5 (5)
			
C5—S1—C2—C3	0.1 (4)	C14—S2—C11—C12	−0.9 (4)
C5—S1—C2—C1	−179.6 (4)	C14—S2—C11—C10	179.8 (4)
C1—C2—C3—C4	179.7 (5)	C10—C11—C12—C13	−179.3 (4)
S1—C2—C3—C4	0.0 (5)	S2—C11—C12—C13	1.4 (5)
C2—C3—C4—C5	−0.2 (6)	C11—C12—C13—C14	−1.3 (6)
C3—C4—C5—C6	−178.6 (4)	C12—C13—C14—C15	178.0 (4)
C3—C4—C5—S1	0.3 (5)	C12—C13—C14—S2	0.6 (5)
C2—S1—C5—C4	−0.2 (4)	C11—S2—C14—C13	0.2 (4)
C2—S1—C5—C6	178.6 (4)	C11—S2—C14—C15	−177.2 (4)
C4—C5—C6—C7	−178.2 (4)	C13—C14—C15—C16	−178.8 (4)
S1—C5—C6—C7	3.1 (7)	S2—C14—C15—C16	−1.8 (7)
C5—C6—C7—C9	−180.0 (4)	C14—C15—C16—C18	175.5 (4)
C5—C6—C7—C8	1.8 (7)	C14—C15—C16—C17	−1.9 (7)
C6—C7—C8—N1	−151 (29)	C15—C16—C17—N3	−92 (17)
C9—C7—C8—N1	30 (29)	C18—C16—C17—N3	91 (17)
C6—C7—C9—N2	6 (43)	C15—C16—C18—N4	21 (69)
C8—C7—C9—N2	−175 (100)	C17—C16—C18—N4	−161 (68)

### Hydrogen-bond geometry (Å, º)

**Table:**

*D*—H···*A*	*D*—H	H···*A*	*D*···*A*	*D*—H···*A*
C3—H3*A*···N2^i^	0.93	2.52	3.439 (6)	168
C6—H6*A*···N3^ii^	0.93	2.52	3.434 (6)	169
C12—H12*A*···N4^iii^	0.93	2.60	3.518 (6)	171
C15—H15*A*···N1^i^	0.93	2.51	3.430 (6)	170

Symmetry codes: (i) *x*, *y*, *z*+1; (ii) *x*+1, *y*−1, *z*−1; (iii) *x*, *y*, *z*−1.
